# A nanocarrier system that potentiates the effect of miconazole within different interkingdom biofilms

**DOI:** 10.1080/20002297.2020.1771071

**Published:** 2020-06-07

**Authors:** Laís Salomão Arias, Jason L Brown, Mark C Butcher, Christopher Delaney, Douglas Roberto Monteiro, Gordon Ramage

**Affiliations:** a Department of Preventive and Restorative Dentistry, São Paulo State University (Unesp), School of Dentistry, Araçatuba, São Paulo, Brazil; b Oral Sciences Research Group, Glasgow Dental School, School of Medicine, Dentistry and Nursing, College of Medical, Veterinary and Life Sciences, University of Glasgow, Glasgow, UK; c Graduate Program in Dentistry (GPD - Master's Degree), University of Western São Paulo (UNOESTE), São Paulo, Brazil

**Keywords:** Biofilms, interkingdom, antimicrobials, miconazole, nanocarriers

## Abstract

**Background:**

Novel and new therapeutic strategies capable of enhancing the efficacy of existing antimicrobials is an attractive proposition to meet the needs of society.

**Objective:**

This study aimed to evaluate the potentiating effect of a miconazole (MCZ) nanocarrier system, incorporated with iron oxide nanoparticles (IONPs) and chitosan (CS) (IONPs-CS-MCZ). This was tested on three representative complex interkingdom oral biofilm models (caries, denture and gingivitis).

**Materials and methods:**

The planktonic and sessile minimum inhibitory concentrations (MICs) of IONPs-CS-MCZ against different *Candida albicans* strains were determined, as well as against all represented bacterial species that formed within the three biofilm models. Biofilms were treated for 24 hours with the IONPs-CS nanocarrier system containing MCZ at 64 mg/L, and characterized using a range of bioassays for quantitative and qualitative assessment.

**Results:**

MIC results generally showed that IONPs-CS-MCZ was more effective than MCZ alone. IONPs-CS-MCZ also promoted reductions in the number of CFUs, biomass and metabolic activity of the representative biofilms, as well as altering biofilm ultrastructure when compared to untreated biofilms. IONPs-CS-MCZ affected the composition and reduced the CFEs for most of the microorganisms present in the three evaluated biofilms. In particular, the proportion of streptococci in the biofilm composition were reduced in all three models, whilst *Fusobacterium* spp. percentage reduced in the gingivitis and caries models, respectively.

**Conclusion:**

In conclusion, the IONPs-CS-MCZ nanocarrier was efficient against three *in vitro* models of pathogenic oral biofilms, showing potential to possibly interfere in the synergistic interactions among fungal and bacterial cells within polymicrobial consortia.

## Introduction

Oral diseases are globally prevalent, and the misdiagnosis or non-treatment of such diseases can result in systemic complications. Gingivitis for example is considered by many a common and easy to treat oral inflammatory disease, but when left untreated can lead to destructive periodontitis, a disease that affects 10% of global population [1]. According to World Health Organization (WHO) surveys, dental caries is the most common disease in humans, affecting 49 to 83.4% of children [1]. Similarly, the prevalence of denture stomatitis has also been increasing in elderly people and denture wearers, as well as immunocompromised patients, and with a drive to maintain natural dentition we have seen a rise in partial dentures in younger adult patients [2–4]. These biofilm-mediated diseases can all be easily prevented with effective self-directed oral hygiene measures (biofilm disruption and chemical disinfection), yet unnecessary risk related to human behavior frequently leads to oral disease requiring antimicrobial intervention. For effective treatment and/or control of different oral biofilms, this requires a tacit understanding of the nature of the causative oral microbiome in order to recapitulate these interactions *in vitro*. Existing biofilm models often fail to account for polymicrobiality, a characteristic associated with the pathogenesis of gingivitis, caries and denture stomatitis [5,6]. Instead, commercial models for drug discovery rely on simplified mono-species biofilms that provide an inferior challenge to antimicrobial therapies. Importantly, and frequently, *C andidaalbicans* participates in inter-kingdom interactions as an opportunistic fungus whose association with different bacteria favours synergized tolerance to conventional treatment [7–10]. This yeast is capable of reducing the oxygen tension in the biofilm and providing growth stimulatory factors that allows a synergistic relationship with bacteria, also supporting the growth of early and later colonizers of oral biofilms [6,8]. *C. albicans* can also physically interact with a number of oral bacterial species such as oral streptococci, *Fusobacterium nucleatum* and *Porphyromonas gingivalis* through well-characterized physical surface protein receptor interactions between bacterial and fungal cells [7,11,12]. As the number of studies on *C. albicans* increases, its physical and metabolic role within the oral microbiome, and inter-dependencies, are beginning to be better understood. Thus, *in vitro* models investigating the development of novel antimicrobial therapies warrant the use of polymicrobial interkingdom biofilms containing fungal and bacterial species to more accurately assess new and novel antimicrobial strategies.

In this current era of antimicrobial resistance, innovating treatments and the discovery and administration of novel antimicrobials is becoming increasingly problematic. The constant exposure of microorganisms to these antimicrobials, even at low levels, can lead to mutations and new resistance mechanisms [13]. Azoles are considered a major problem in this sense, with oral candidiasis including denture stomatitis frequently treated with azole antifungals [14]. In addition, use of broad spectrum antibiotics including azoles such as metronidazole have been reported for treatment of periodontal lesions resulting from mixed bacterial and fungal infections [15]. Miconazole (MCZ) has also proven to be a good antimicrobial choice since its broad-spectrum activity effects against both fungi and different bacteria species [16]. Such activity is clinically desirable for a polymicrobial clinical challenge, but clinical and laboratory-based reports of microbial resistance and azole cross resistance alert us to the risk of over-reliance on these treatments [10,17–19]. Thus, alternative strategies such as the use of drug nanocarriers which enhance the action of conventional drugs by facilitating their delivery to target cells and overcoming the biofilm physical barriers warrant further consideration. These systems aim to reduce the concentration of drug required, exposure time and increase the efficacy of the drug against the microorganisms [20,21]. To this end, iron oxide magnetic nanoparticles (IONPs) have recently been studied for different clinical applications, including hyperthermia, magnetic resonance imaging, tissue repair and drug delivery [21]. More recently, *in vitro* studies have described IONP-based nanosystems that possess anti-biofilm purposes, whose nano- and magnetic properties promise to meet the optimization needs of antimicrobial treatments, interfering with the composition of pathogenic biofilms [22,23]. Therefore, the aim of the present study was to investigate the effect of a recently developed MCZ nanocarrier based on IONPs and chitosan (CS) [24], on the composition of three *in vitro* biofilm models representative of pathogenic interkingdom oral biofilms. This is the first study to show that a nanosystem based on IONPs and CS can efficiently carry miconazole and are effective against pathogenic oral polymicrobial interkingdom biofilms *in vitro*.

## Materials and methods

All biofilm work for this study and preparation of the manuscript was carried out in accordance with the minimum information guidelines specified for biofilm formation in microplates [25].

### Assembly and characterization of the IONPs-CS-MCZ nanocarrier

A stock solution of the nanocarrier was prepared by the mixture of equal volumes of a IONPs colloidal suspension (700 mg/L) and CS (700 mg/L) followed by the MCZ solubilization (500 mg/L) into the *core-shell* compound IONPs-CS, as previously described for a chlorhexidine-carrier nanosystem [22]. As before, the colloidal suspension of IONPs was kindly provided by *nChemi* (Sao Paulo, Brazil). Development of this novel IONPs-CS-MCZ system is detailed elsewhere [24].

### Strains and growth conditions

Twelve strains of *C. albicans* were utilized in the current study, including 10 clinical oral isolates (BC020, BC023, BC037, BC038, BC039, BC117, BC136, BC145, BC044, BC146) from the Oral Sciences Research Group, University of Glasgow, Glasgow, UK, and a key laboratory reference strain (ATCC 10,231). Stock cultures stored in Microbank^TM^ beads (Pro-lab Diagnostics, UK) at −80°C were revived on Sabouraud dextrose agar (SAB agar). For the MIC and biofilm assays, a loopful of *Candida* colonies was suspended in 10 mL of Yeast Extract Peptone Dextrose (YPD) medium using a disposable 10 µL inoculating loop (Thermo-Fisher, UK). Broths were cultured aerobically overnight at 30°C, constantly shaken at 120 rpm. Following centrifugation, the fungal pellet was washed twice in phosphate buffered saline (PBS) then the suspension adjusted with the aid of a haemocytometer to 2 × 10^4^ cells/mL, 1 × 10^6^ cells/mL and 1 × 10^7^ cells/mL for planktonic, mono-culture, and polymicrobial biofilm evaluations, respectively.

For the multi-species biofilm models, the following bacterial strains stored on Microbank^TM^ beads at −80°C were used: *Streptococcus mitis* NCTC 12,261, *Streptococcus oralis* NCTC 11,427, *Streptococcus intermedius* DSM 20,573, *Streptococcus mutans* NCTC 10,449, *Veillonella dispar* NCTC 11,831, *Actinomyces naeslundii* DSM 17,233, *Lactobacillus casei* DSMZ 20,011, *Lactobacillus zeae* DSM 20,178, *Rothia dentocariosa* DSM 43,762, *Fusobacterium nucleatum* ATCC 10,953 and *Fusobacterium nucleatum vincentii* DSM 19,507. *Streptococcus* and *Rothia* species were revived on Columbia Blood Agar base containing 10% defibrinated horse blood (CBA; Sigma–Aldrich, UK) overnight in 5% CO_2_. Strict and facultative anaerobic strains (*V. dispar, A. naeslundii, F. nucleatum, F. nucleatum vicentii*) were cultivated on Fastidious Anaerobe Agar base containing 10% defibrinated horse blood (FAA; Sigma–Aldrich, UK) in an anaerobic incubator (Don Whitley Scientific Limited, UK) for 2–3 days, while *Lactobacillus* strains were propagated on De Man Rogosa Sharpe Agar (MRS agar; Sigma–Aldrich, UK) overnight in 5% CO_2_. The temperature of incubation for all aforementioned bacterial strains was 37°C. Following growth on solid agar, a loopful from each bacterial strain was suspended in 10 mL of specific media (Tryptic Soy Broth (TSB; Sigma–Aldrich, UK) for *Streptococcus* species, Brain Heart Infusion broth (BHI; Difco, UK) for *A. naeslundii, V. dispar* and *R. dentocariosa*, Schaedler’s anaerobic broth (SCH; Oxoid, UK) for *Fusobacterium* and MRS broth for *Lactobacillus* species) and incubated overnight at 37°C. After the incubation period, the bacterial cells were washed twice in PBS then concentration was adjusted to 1 × 10^8^ cells/mL using a spectrophotometer. Absorbance readings at A_550_ _nm_ that equate to 1 × 10^8^ cells/mL were determined using serial dilutions of pure cultures and Miles and Misra colony counting technique [26].

### Determination of the MICs

MIC testing was employed to determine the MCZ and IONPs-CS-MCZ MICs for planktonic (pMIC) and sessile (sMIC) cells of the studied fungal and bacterial strains. Briefly, for planktonic cells, 100 µL of standardized cell suspensions (2 x 10^4^ cells/mL for *Candida* and 2 × 10^5^ cells/mL for bacterial cells) in the appropriate media for each microorganism described above were pipetted in clear bottom round-bottom wells of 96-well plates (Corning, UK) containing 100 µL of a 2-fold serial dilution of MCZ and IONPs-CS-MCZ with concentration ranging from 0.5–256 mg/L, and the pMIC values were visually determined after 24–48 hours. For this, the lowest concentration to visually prevent growth of the microorganisms was deemed the pMIC.

To determine the sMIC, 200 µL of the microbial inoculum (1 x 10^6^ cells/mL for fungal cells and 1 × 10^7^ for bacterial cells) were pipetted in flat-bottom wells of 96-well clear bottom plates (Corning, UK) and biofilm allowed to form for 24 hours at 37°C. The biofilms were treated with 2-fold serial dilutions of MCZ and IONPs-CS-MCZ ranging from 0.5–256 mg/L and incubated at 37°C under specific aerobic or anaerobic conditions dependent on microbial species. After 24 hours, the resulting biofilms were gently washed with PBS prior to the addition of 100 µL of XTT [2,3-bis(2-methoxy-4-nitro-5-sulfophenyl)-2 H-tetrazolium-5-carboxanilide salt] (Sigma Aldrich, UK). After 2 hours of incubation at 37°C, the absorbance values were read at 490 nm and the sMIC_50_ and sMIC_80_ were considered the concentrations that lead respectively to 50% and 80% reduction of XTT readings, when compared to the untreated positive control. All MIC tests were carried out on two separate occasions in quadruplicate wells of a 96-well plate. For all experiments, appropriate controls were included as follows; positive controls minus antimicrobial intervention and negative controls minus inoculum were included on each plate in quadruplicate.

### Multi-species biofilm models and treatment

To assess the effect of IONPs-CS-MCZ on pathogenic biofilms, three different *in vitro* multi-species biofilm models were tested, representative of caries, denture and gingivitis developed as previously described [4,27,28]. For all biofilm models, the bacterial and fungal cells were adjusted to 1 × 10^7^ cells/mL in Todd Hewitt Broth (THB) medium supplemented with 0.01 mg/mL hemin and 2 µg/mL menadione [29]. Biofilms were formed directly on the bottom of wells of 24-well plates (Corning, UK) for XTT reduction and biomass quantification assays, or on 13 mm Thermanox coverslips (Thermo-Fisher, UK) placed in 24-well plates for CFU counting, qPCR assays and SEM analysis.

Biofilm development for all models used followed similar protocols as described previously [4,27,28,30]. Briefly, biofilm formation consisted of inoculating *Streptococcus* species and *C. albicans*(500 µL) on the first day to promote primary colonization, followed by the addition of the remaining species on the second day. In brief, for the gingivitis model, *C. albicans, S. mitis, S. oralis* and *S. intermedius* were added on day 1 and *V. dispar, A. naeslundii, F. nucleatum* and *F. nucleatum vicentii* were added on day 2. For the denture model, *C. albicans, S. mitis, S. oralis and S. intermedius* were added on day 1 and *V. dispar, A. naeslundii, L. casei, L. zeae* and *R. dentocariosa* were added on day 2. Finally, for dental caries model, *C. albicans* and *S. mutans* were added on day 1 and *V. dispar, A. naeslundii, F. nucleatum* and *L. casei* were added on day 2. All biofilms were then matured anaerobically for an additional 4 days, and the culture media was replenished every 24 hours. Afterwards, the stock solution of the IONPs-CS-MCZ nanocarrier was diluted in THB supplemented media to the concentration of 64 mg/mL of MCZ, which was used to treat the biofilms for 24 hours. Biofilms were also treated with MCZ only at the same concentration, minus nanocarrier delivery. Biofilms minus treatment (containing media only) were considered as controls.

### Semi-quantitative biofilm analyses

After the treatment period, the biofilms were gently washed three times with PBS then incubated with 250 µL of XTT. The plates were then protected from light and incubated for 2 hours at 37°C. Next, 100 µL of each well’s content was transferred to 96-well plates and the absorbance read at 490 nm to determine the biofilms’ metabolic activity. To quantify biofilm biomass, the crystal violet method was applied, as described elsewhere [10]. Briefly, biofilms received 250 µL of 0.05% w/v crystal violet for 20 minutes. Then, the remaining dye was removed, and the biofilms were washed with water to remove any excess dye. The microtiter plates were dried at room temperature and biofilms de-stained with 99% ethanol. Finally, 100 µL of each wells’ content were transferred to 96-well microtiter plates and the results were read at 570 nm. For all XTT and CV assays on polymicrobial biofilms, experiments were done on three independent occasions in triplicate in 24-well plates. Appropriate controls minus microbial inoculum were included on each plate in triplicate.

To analyze the biofilms ultrastructure after exposure to MCZ only or the IONPs-CS-MCZ nanocarrier, the biofilms were prepared for SEM analysis as reported previously [31]. Briefly, the resulting biofilms were washed with PBS and fixed in a solution containing 2% glutaraldehyde, 2% paraformaldehyde, 0.15% alcian blue and 0.15 M sodium cacodylate (pH 7.4), and further processed for SEM analysis. The samples were sputter-coated with gold and viewed under a JEOL JSM-6400 scanning electron microscope (JEOL Ltd, UK) at a magnification of 1,000 x and 3,500 x, respectively.

### Quantitative biofilm analyses

Following treatment, the coverslips containing biofilms were gently washed three times with PBS, removed from the wells and transferred into bijoux tubes containing 1 mL of PBS for sonication at 35 kHz for 10 min [30,32] to facilitate the disaggregation of biofilm cells. For CFU counting, the Miles and Misra technique was employed [26]. Serial dilutions were made in PBS then plated on specific agar media (SAB for *Candida*, and CBA and FAA supplemented with 0.025 mg/mL of Amphotericin B for aerobic and anaerobic growth respectively). The CFUs were counted after 48 hours incubation at 37°C for CBA and FAA plates, and 30°C for 48 h for SAB plates.

A qPCR viability method was used to count the microbial cells and determine the composition of the biofilm models, as previously described [4,30]. In short, the sonicated samples from above were split into 2 Eppendorf tubes. For each group, one of the Eppendorfs received 50 µM of propidium monoazide (PMA; Sigma-Aldrich, UK) to quantify the viable cells, prior to incubation of all samples in the dark for 10 minutes to allow uptake and intercalation of the PMA dye in double stranded DNA (dsDNA) of compromised cells. Next, both sets of samples were exposed to 650 W halogen light for 5 min; this exposure to visible light initiates a covalent linkage to form in the dsDNA inhibiting DNA amplification, allowing for clear quantification of dead and viable cells via real-time quantitative polymerase chain reaction (qPCR). The DNA was then extracted from the samples by using the QIAmp mini DNA Extraction Kit (Qiagen, UK), according to the manufacturer’s recommendations, and qPCR was employed to quantify the viable cells of the biofilms. For qPCR,1 µL of sample DNA was added to a mastermix solution containing 10 µL of SYBR GreenER^TM^ (Thermo-Fisher, UK), 7 µL of UV-treated RNase-free water and 1 µL of 10 µM forward/reverse primers for each microbial genus or species. Table 1 displays the primers used in the study [4,27,28]. A total volume of 20 μl was added to MicroAmp fast-optical 96-well 0.1 ml reaction plates (Applied Biosystems, USA) and loaded into the StepOnePlus™ real time system (Applied Biosystems, USA). The following thermal cycle was used: 95°C for 2 minutes, 40 amplification cycles of 95°C for 3 seconds followed by 55°C for 30 seconds. All samples were run in duplicate. Colony forming equivalents (CFEs) were quantified using a standard curve of bacterial and fungal CFUs ranging from 1 × 10^3^ to 10^8^ CFU/mL. For CFU counts and qPCR analyses, experiments were completed in triplicate on two independent occasions. Appropriate controls minus inoculum were included for each experiment to assess for microbial contamination of the media.

**Table 1.** Bacterial and fungal primer sequences for real time PCR.

Target	Primer sequence (5ʹ – 3ʹ)	Reference
*C. albicans*	F – GGGTTTGCTTGAAAGACGGTA	[30]
	R – TTGAAGATATACGTGGTGGACGTTA	
*Streptococcus spp.*	F – GATACATAGCCGACCTGAG	[30]
	R – CCATTGCCGAAGATTCC	
*A. naeslundii*	F – GGCTGCGATACCGTGAGG	[30]
	R – TCTGCGATTACTAGCGACTCC	
*V. dispar*	F – CCGTGATGGGATGGAAACTGC	[30]
	R – CCTTCGCCACTGGTGTTCTTC	
*Fusobacterium spp.*	F – GGATTTATTGGGCGTAAAGC	[30]
	R – GGCATTCCTACAAATATCTACGAA	
*L. casei*	F – TGCACTGAGATTCGACTTAA	[27]
	R – CCCACTGCTGCCTCCCGTAGGAGT	
*L. zeae*	F – TGCATCGTGATTCAACTTAA	[4]
	R – CCCACTGCTGCCTCCCGTAGGAGT	
*R. dentocariosa*	F – GGGTTGTAAACCTCTGTTAGCATC	[4]
	R – CGTACCCACTGCAAAACCAG	

### Statistical analysis

All microbiological assays were performed in triplicate on at least two independent occasions. All graphs were compiled and where appropriate, normally distributed data analysed by one-way ANOVA with Tukey’s post-hoc to compare the means of more than two group. A significance level was determined at 5%, using the statistical program GraphPad Prism (version 7; La Jolla, CA, USA).

## Results

### *Determination of the MICs of* Candida *and bacterial strains*

The results of pMICs for all *C. albicans* strains revealed the same susceptibility pattern to MCZ and IONPs-CS-MCZ, with values ranging from 2 to 4 mg/L (Table 2). For sMIC results, it was possible to observe lower MIC values for the biofilms in response to the nanocarrier compared to the MCZ, for both sMIC_50_ and sMIC_80_, respectively. The analysis of susceptibility of oral strains to MCZ showed that concentrations of 16 to 128 mg/L (sMIC_80_) were required to reduce the metabolism of the biofilms (Table 2). As for the nanocarrier, lower concentrations were required for all oral *C. albicans* strains assessed under the same conditions (sMIC_80_). The concentrations of MCZ required in the nanosystem ranged from 0.25 to 16 mg/L for all strains. A similar pattern was observed for the reference strain of *C. albicans* (ATCC 10,231), in which sMIC_80_ was 64 mg/L for MCZ but 32 mg/L for MCZ in the IONPs-CS-MCZ system (Table 2).

**Table 2.** Values of minimum inhibitory concentration (MIC) of miconazole (MCZ) and MCZ nanocarrier (IONPs-CS-MCZ) for planktonic (pMIC) and sessile (sMIC_50_ and sMIC_80_) cells of 10 different oral *Candida albicans* strains and one type strain, ATCC 10,231. MIC values calculated from two independent experiments with quadruplicate technical replicates.

	MCZ (mg/L)			IONPs-CS-MCZ (mg/L)		
*C. albicans* strains	pMIC	sMIC_50_	sMIC_80_	pMIC	sMIC_50_	sMIC_80_
BC020	4	4	32	4	0.25	4
BC023	4	16	128	4	4	4
BC037	4	0.25	16	4	0.25	0.25
BC038	4	16	32	4	0.25	16
BC039	4	32	32	4	0.25	8
BC117	4	8	32	4	0.25	1
BC136	4	16	32	4	0.25	2
BC044	2	0.25	32	2	0.25	2
BC145	4	8	32	4	0.25	2
BC146	2	8	32	2	0.25	2
ATCC 10,231	4	16	64	4	2	32

Table 3 shows the MIC values of the *C. albicans* and bacterial strains included in the three studied biofilm models (gingivitis, denture and dental caries). For all bacterial species, MCZ and IONPs-CS-MCZ displayed the same pMIC values, except for *R. dentocariosa*, for which the nanocarrier showed lower values than MCZ alone (>128 mg/L for MCZ only, compared to 8 mg/L for the MCZ in the nanocarrier system). Regarding sMIC_50_, IONPs-CS-MCZ showed similar or slightly lower values compared to MCZ, except for *A. naeslundii*, whose sMIC_50_ value for the nanocarrier was 4 times higher than that found for MCZ alone (16mg/L for MCZ compared to 64 mg/L for IONPs-CS-MCZ). Conversely, the nanocarrier displayed sMIC_80_ values lower than MCZ for all bacterial strains except for *L. casei, L. zeae* and *R. dentocariosa* which were the same values for both treatments. It is noteworthy that treatment of all *Candida* and bacterial strains with nanoparticles only minus MCZ gave no detectable MICs. Taken together, the results from the preceding section indicate that IONPs-CS-MCZ nanocarrier was more effective than MCZ alone against biofilms containing *C. albicans* and most bacterial species investigated in this study.

**Table 3.** Values of minimum inhibitory concentration (MIC) of miconazole (MCZ) and MCZ nanocarrier (IONPs-CS-MCZ) for planktonic (pMIC) and sessile (sMIC_50_ and sMIC_80_) cells of the species included in the different studied biofilm models. All MIC tests were carried out on two separate occasions in quadruplicate wells of a 96-well plate.

		pMIC [mg/L]		sMIC_50_ [mg/L]		sMIC_80_ [mg/L]	
	Biofilm model	MCZ	IONPs-CS-MCZ	MCZ	IONPs-CS-MCZ	MCZ	IONPs-CS-MCZ
*C. albicans* 3153A	C/G/D	4	4	8	<0.5	16	4
*S. mitis* 12,261	G/D	4	4	16	16	>256	64
*S. oralis* 11,427	G/D	4	4	4	4	>256	64
*S. intermedius* 20,573	G/D	<1	<1	64	64	256	64
*S. mutans* 10,449	C	4	4	128	64	256	64
*V. dispar* 11,831	C/G/D	16	16	32	16	>256	32
*A. naeslundii* 17,233	C/G/D	8	8	16	64	256	128
*F. nucleatum* 10,953	C/G	4	4	32	16	32	16
*F. nucleatum vincentii* 10,507	G	2	2	16	4	32	8
*L. casei* 20,011	C/D	16	16	>256	256	>256	>256
*L. zeae* 20,178	D	16	16	>256	64	>256	>256
*R. dentocariosa* 43,762	D	>128	8	32	32	64	64

Note: Capital letters C, G and D represent, respectively, the caries, gingivitis and denture biofilm models.

### Biofilm characterization

**Figure 1.**
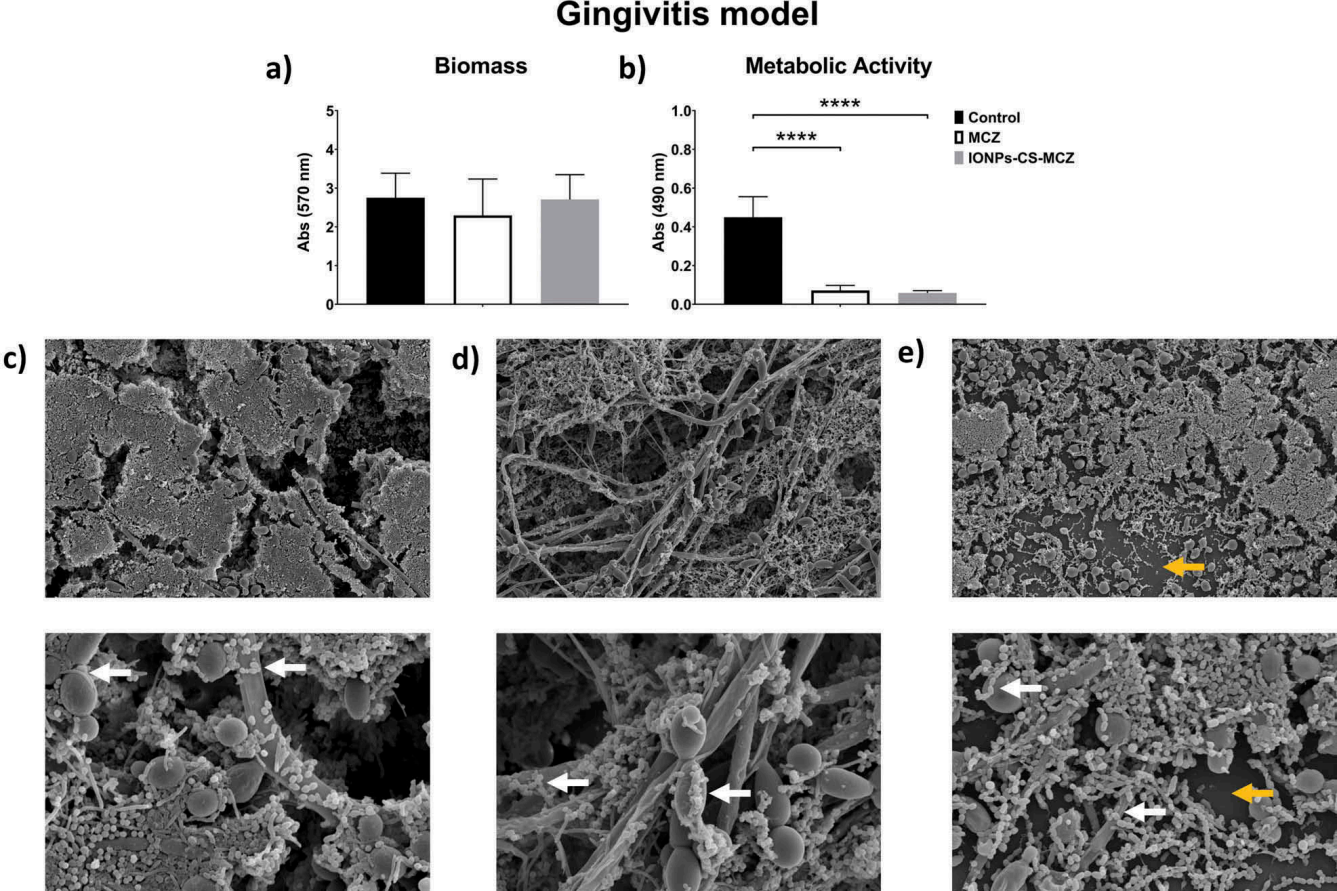
Results of XTT reduction assay (A), quantification of total biomass (B) and scanning electron microscopy (SEM) observation (C) for the gingivitis biofilm model untreated (control) and (D) treated with the miconazole (MCZ) only or (E) nanocarrier containing MCZ at 64 mg/L (IONPs-CS-MCZ) anaerobically for 24 hours. Magnification of the SEM images: 1,000x and 3,500x; Bars: 10 and 5 µm. Significant differences between the groups were calculated by one-way ANOVA with Tukey’s post-hoc test (* p < 0.05, **p < 0.01, *** p < 0.001, **** p < 0.0001). Results shown in A and B representative of a total of 9 values for each treatment e.g., three technical replicates from three separate experiments. White arrows represent adhesion of bacteria to yeasts/hyphae. Yellow arrows highlight the visible coverslip due to loss of biofilm biomass by IONPs-CS-MCZ.

**Figure 2.**
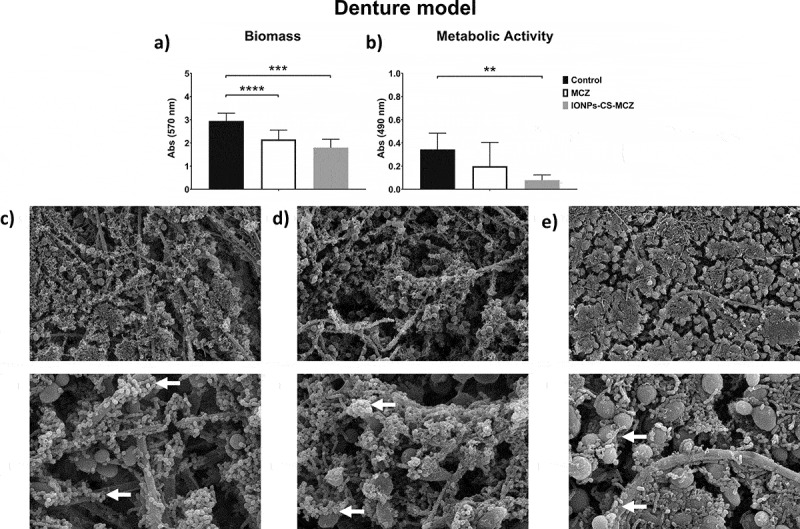
Results of XTT reduction assay (A), quantification of total biomass (B) and scanning electron microscopy (SEM) observation (C) for the denture biofilm model untreated (control) and (D) treated with miconazole (MCZ) only or (E) the nanocarrier containing MCZ at 64 mg/L (IONPs-CS-MCZ) anaerobically for 24 hours. Magnification of the SEM images: 1,000x and 3,500x; Bars: 10 and 5 µm. Significant differences between the groups were calculated by one-way ANOVA with Tukey’s post-hoc test (* p < 0.05, **p < 0.01, *** p < 0.001, **** p < 0.0001). Results shown in A and B representative of a total of 9 values for each treatment e.g., three technical replicates from three separate experiments. White arrows represent adhesion of bacteria to yeasts/hyphae. Yellow arrows highlight the visible coverslip due to loss of biofilm biomass following treatment with IONPs-CS-MCZ.

**Figure 3.**
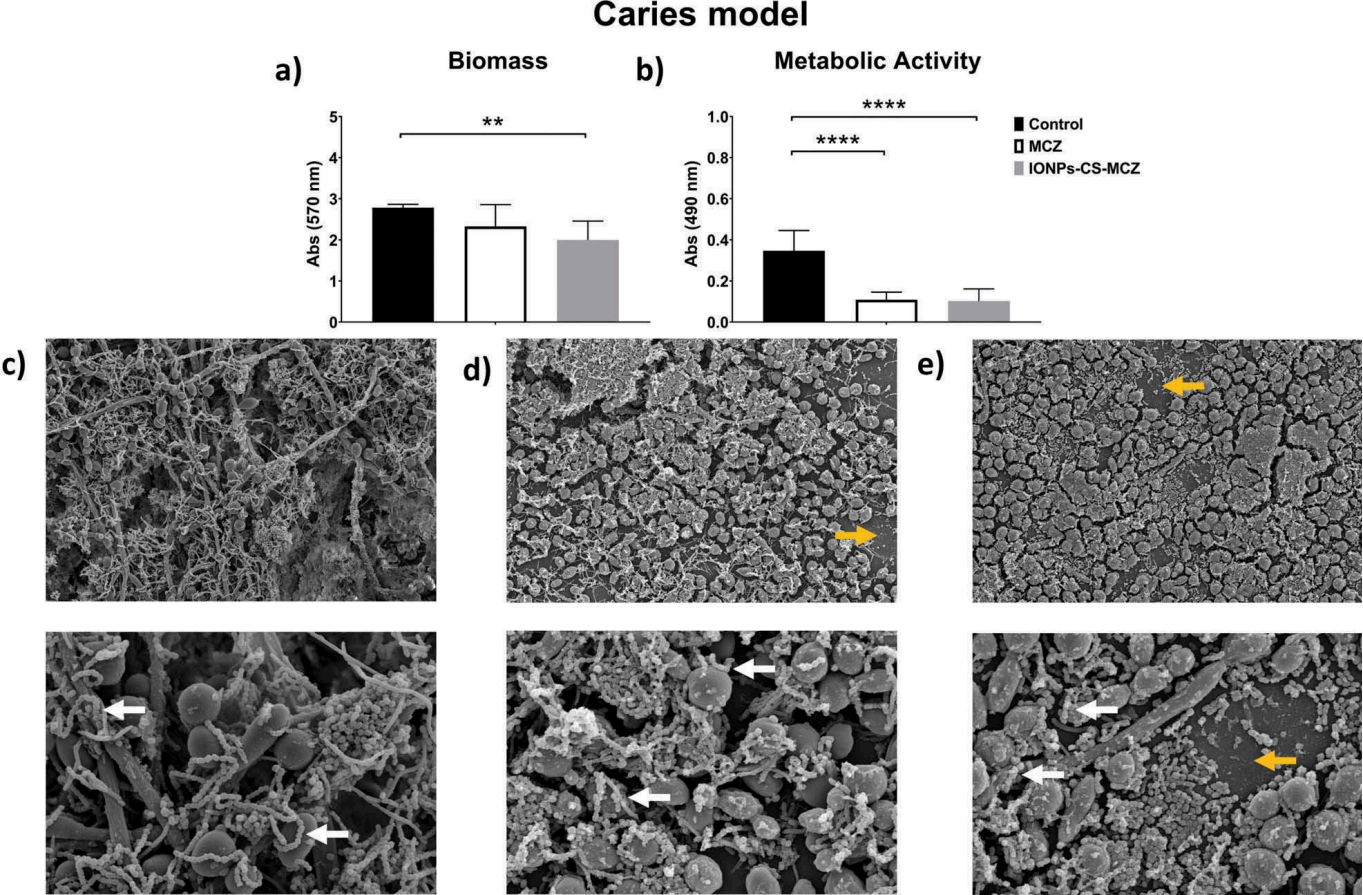
Results of XTT reduction assay (A), quantification of total biomass (B) and scanning electron microscopy (SEM) observation (C) for the caries biofilm model untreated (control) and (D) treated with miconazole (MCZ) only or (E) the nanocarrier containing MCZ at 64 mg/L (IONPs-CS-MCZ) anaerobically for 24 hours. Magnification of the SEM images: 1,000x and 3,500x; Bars: 10 and 5 µm. Significant differences between the groups were calculated by one-way ANOVA with Tukey’s post-hoc test (* p < 0.05, **p < 0.01, *** p < 0.001, **** p < 0.0001). Results shown in A and B representative of a total of 9 values for each treatment e.g., three technical replicates from three separate experiments. White arrows represent adhesion of bacteria to yeasts/hyphae. Yellow arrows indicate areas of coverslip now visible following treatment.

**Figure 4.**
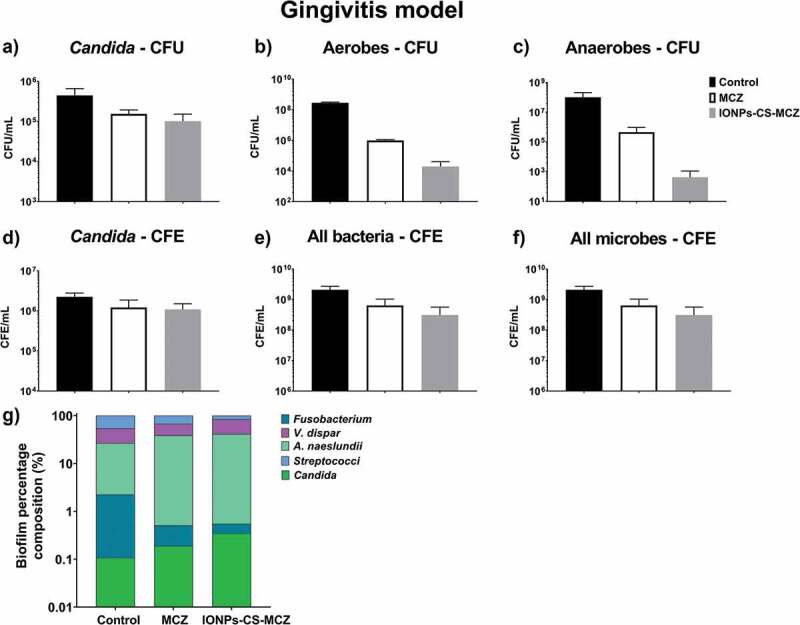
Results of counting of colony forming units for total *Candida* (A), total aerobes (B) and total anaerobes (C) in treated (MCZ only or IONPs-CS-MCZ) biofilms and untreated controls in the gingivitis model. Colony forming equivalents of viable cells of *Candida* (D), total bacteria (E) and total microbes (F) are also shown with biofilm percentage composition represented in panel G. The composition graph y axis has been set to log scale to allow for visualization of all microbial genus/species. Mean percentage proportions for each microorganism are shown in Table 4. Mean CFE/ml values for each organism are shown in Supplementary Table 1. Biofilms were treated anaerobically for 24 hours. Untreated biofilms were cultured under the same conditions minus drug treatment. Results shown representative of a total of 6 values (three technical replicates from two separate experiments).

For all three evaluated oral models, significant reductions in the metabolic activity and total biomass were observed for all biofilms after exposure to IONPs-CS-MCZ compared to the control group, with values ranging from 28 to 86.6% (Panels A and B in Figures 1, 2 and 3), except for gingivitis model biomass, which did not significantly differ from the control group (Figure 1A). However, no statistical differences were seen in the metabolic activity or biomass between the MCZ- and nanocarrier- treated biofilms. SEM analyses highlighted that the control group showed a robust scaffold with dense network of interconnected cells. Bacteria were visibly adhered to the yeast and hyphae of *C. albicans* (denoted by white arrows in Figures 1C, 2C and 3C). Such adhesion between bacteria and fungi was still visible following treatment with MCZ only and the nanocarrier containing the drug (Figure 1D and 1E, 2D and 2E and 3D and 3E). Interestingly, exposure to the nanocarrier resulted in a reduction in biofilm coverage with certain areas of the coverslips now visible in the gingivitis and caries model (denoted by yellow arrows in Figures 1E and 3E). A similar trend was seen for the MCZ only treatment in the caries model (Figure 3D). However, biofilm coverage was relatively comparable between the untreated, MCZ- and nanocarrier-treated samples for the denture biofilm model with no obvious change in the biofilm coverage or thickness (Figure 2C and 2E). In all models less hyphae were present in IONPs-CS-MCZ-treated biofilms compared to untreated controls, with *Candida* visible mostly in the form of yeast cells (Figures 2E, 3E and 4E).

### Quantitative biofilm assessment

Figure 4 shows the effect of the nanocarrier IONPs-CS-MCZ on the gingivitis biofilm model, compared with the MCZ treatment alone, and negative control untreated biofilms. Overall, CFU and CFE quantifications revealed that the number of all microorganisms treated with MCZ only and IONPs-CS-MCZ were reduced in comparison to the control group (Figure 4 and Supplementary Figure 1). In this biofilm model, the effects of MCZ alone and MCZ in the nanosystem were more evident against the bacterial cells and less so against the *Candida* cells. The compositional analysis of the gingivitis biofilm model showed that treatment with MCZ and with the nanocarrier altered the composition of the biofilm (Figure 4G, Table 4 and Supplementary Table 1). The greatest change occurred for the *Streptococcus* spp., which was the most predominant consortia in the biofilm from the control group (representing ~46% of biofilm total cells), second most dominant in the MCZ-treated group (~32% of the total), yet only the third most prevalent in the nanosystem-treated group (~15% of the total).

**Table 4.** Mean % composition values for each microorganism from the three different biofilm models (gingivitis, denture and dental caries). Mean values taken from a total of 6 colony forming equivalent values for all microorganisms in each biofilm model e.g., three technical replicates from two experiments.

		Gingivitis model			Denture model			Dental caries model		
		Control	MCZ	Nanocarrier	Control	MCZ	Nanocarrier	Control	MCZ	Nanocarrier
	Total CFE/mL	2.1x 10^9^	4.4 x 10^8^	3.1 x 10^8^	6.8 x 10^8^	5.5 x 10^7^	6.4 x 10^6^	5.9 x 10^8^	1.4 x 10^8^	6.2 x 10^7^
Percentage composition (%)	***Candida***	0.1%	0.2%	0.3%	2.3%	14.4%	6.5%	4.6%	7.4%	35.4%
	***Streptococci***	46.1%	32.3%	15.4%	49.4%	10.6%	9%	17.3%	53.1%	0.8%
	***V. dispar***	27.3%	28.8%	43.6%	25.9%	21.7%	4.9%	44.8%	10.9%	33.4%
	***A. naeslundii***	24.4%	38.3%	40.5%	21.0%	24.2%	58.0%	31.6%	25.3%	13.2%
	***Fusobacterium***	2.1%	0.32%	0.2%	n/a	n/a	n/a	0.6%	0.05%	0.04%
	***L. casei***	n/a	n/a	n/a	0.4%	5.1%	3.8%	1.1%	3.3%	17.1%
	***L. zeae***	n/a	n/a	n/a	0.9%	23.9%	17.6%	n/a	n/a	n/a
	***R. dentocariosa***	n/a	n/a	n/a	0.1%	0.02%	0.2%	n/a	n/a	n/a

**Figure 5.**
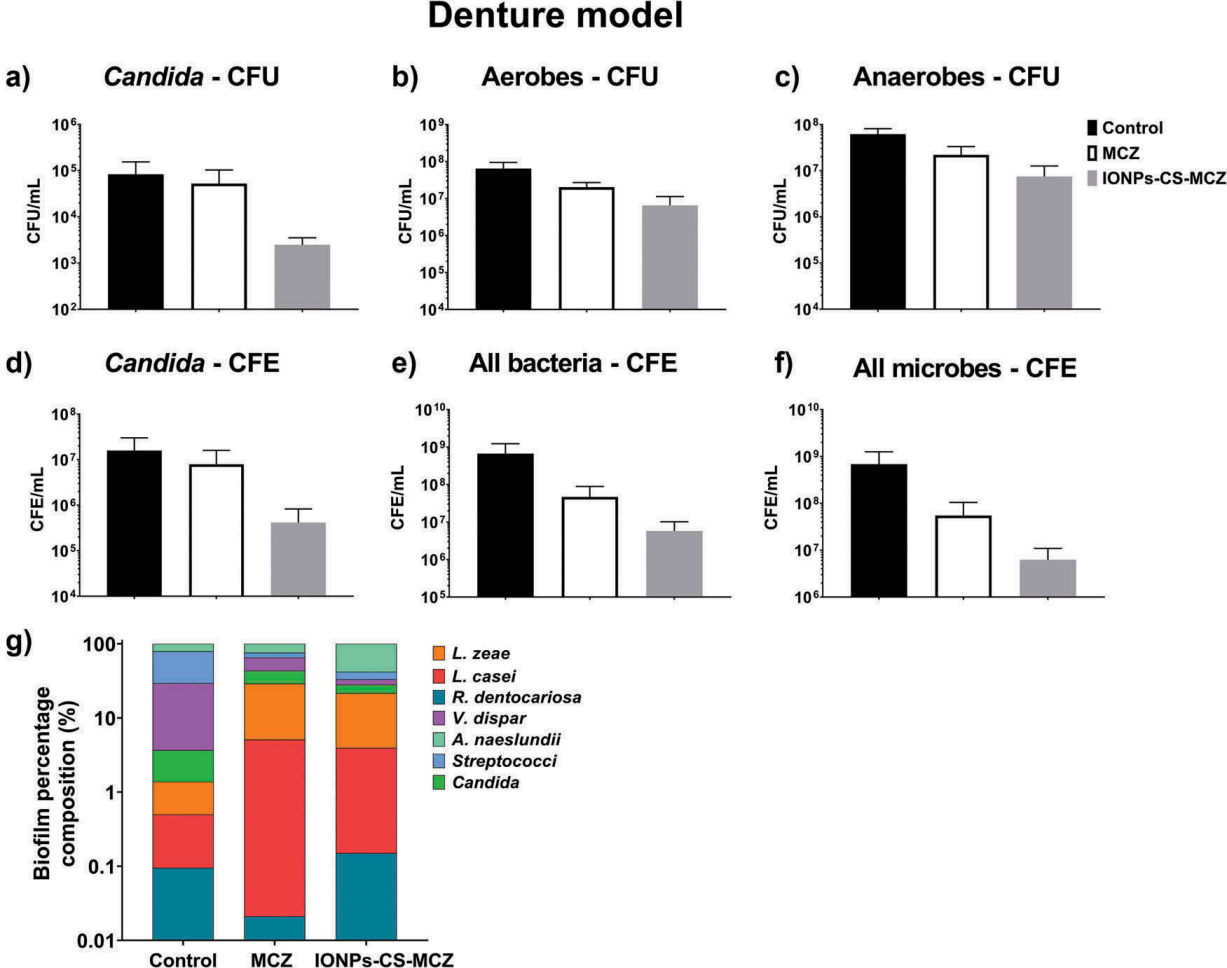
Results of counting of colony forming units for total *Candida* (A), total aerobes (B) and total anaerobes (C) in MCZ- or nanocarrier-treated and untreated controls in the denture biofilm model. Panels D, E and F show the colony forming equivalents of viable cells for *Candida*, total bacteria and total microbes identified using qPCR, respectively. Biofilm percentage composition represented in panel G with % composition and raw CFE/ml values shown in Table 4 and Supplementary Table 1. The composition graph y axis has been set to log scale to allow for visualization of all microbial genus/species. Mature biofilms were treated anaerobically for 24 hours. Untreated biofilms were cultured under the same conditions minus drug treatment. Results shown representative of a total of 6 values (three technical replicates from two separate experiments).

In the denture model, the number of CFUs and CFEs of all species after treatment with MCZ and the nanocarrier decreased compared to the control group (Figure 5 and Supplementary Figure 2), with the exception of the two *Lactobacillus* species whose numbers did not change in the biofilm following treatment with MCZ only. However, treatment of the biofilm with the nanocarrier reduced the numbers of *L. casei* and *L. zeae* by 0.5 log and 1.0 log, respectively, compared to the untreated control biofilm (Supplementary Figure 2E and F). Unlike the previous biofilm model, the nanosystem reduced the amount of *Candida* cells in the denture biofilm, as seen using both quantitative techniques (Figure 5A and 5D). The proportion of microorganisms within the denture biofilm changed following treatment with MCZ and IONPs-CS-MCZ (Figure 5G, Table 4 and Supplementary Table 1). Interestingly, the numbers of *Streptococcus* spp. and *V. dispar* (first [~49%] and second [~26%] most prevalent genus/species in the untreated biofilms) were clearly reduced and replaced by *A. naeslundii* (~60%) and *L. zeae* (~18%) as the most prevalent species in the final composition of the nanosystem-treated biofilms (Figure 5G). Similarly, for the MCZ only treated biofilms, *Streptococcus* spp. and *V. dispar* proportions were reduced to ~11% and ~22%, respectively, replaced with *A. naeslundii* (~24%) and *L. zeae* (~24%) as the most dominant species following treatment.

**Figure 6.**
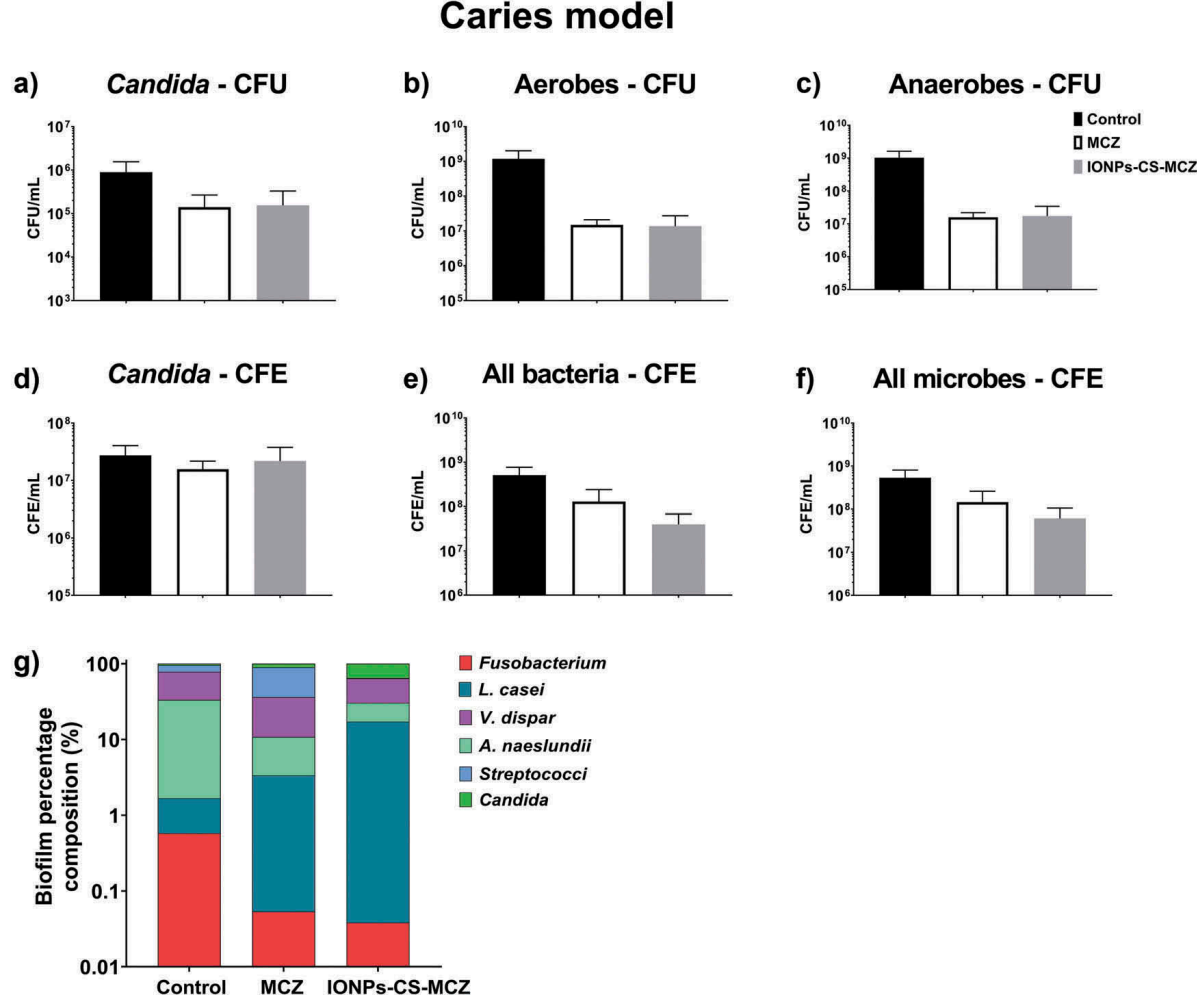
Results of counting of colony forming units for total *Candida* (A), total aerobes (B) and total anaerobes (C) in MCZ- or nanocarrier-treated and untreated controls in the caries biofilm model. Colony forming equivalents of viable *Candida* and total bacteria cells are shown in panels D (*Candida*), E (bacteria) and F (all microbes). Biofilm percentage composition in treated and untreated controls represented in panel G, shown with Log scaling to depict all microbial genus/species. Percentage composition and raw CFE/ml for each microorganism is shown in Table 4 and Supplementary Table 1. Biofilms were treated anaerobically for 24 hours. Untreated biofilms were cultured under the same conditions minus drug treatment. Results shown representative of a total of 6 values (three technical replicates from two separate experiments).

Finally, analyzing the caries model biofilm, CFU data showed that yeast cells were reduced in number when exposed to MCZ only and the nanocarrier in comparison to the control group (Figure 6A). However, the same pattern was not observed for the CFE counting, as *C. albicans* cells reductions were comparable (Figure 6D). However, for all the bacterial species included in this model, there were reductions in the CFU and total CFE counts (Figure 6B, 6C and 6E and Supplementary Figure 3), with the exception for the CFE of streptococci following treatment with MCZ only (Supplementary 3A), and for the CFE of *L. casei* for both treatments (Supplementary Figure 3E). These results are also reflected in the percentages of the biofilm composition. *C. albicans* and *L. casei* increased their prevalence in the total number of cells for MCZ- and nanocarrier-treated biofilms (Figure 6G, Table 4 and Supplementary Table 1). Furthermore, the proportion of *Streptococcus spp*. in the biofilm treated with IONPs-CS-MCZ was reduced considerably when compared to the control (~1% for treated compared to ~17% for untreated).

Interestingly, the proportion of *C. albicans* increased in the final composition of all biofilm models in the MCZ- and IONPs-CS-MCZ-treated biofilms (~0.1% to ~0.2% and ~0.3%, respectively in the gingivitis model, ~2.3% to ~14.4% and ~6.5%, respectively in the denture model and ~4.6% to ~10.9% and ~35.4%, respectively in the caries model). This is suggestive that *Candida* is generally more tolerant to MCZ or IONPs-CS-MCZ treatment in polymicrobial biofilms than most bacterial species, although proportions of *C. albicans* were relatively low in the gingivitis model (~0.1–0.3%). Taken together, the results from this study suggest that the nanocarrier system may improve the efficacy of MCZ in polymicrobial communities, albeit by targeting specific genus or species within the consortia. Of note, *Streptococcus spp*. numbers were reduced by 1.5–2.5 log in all biofilm models following treatment with IONPs-CS-MCZ.

## Discussion

**Figure 7.**
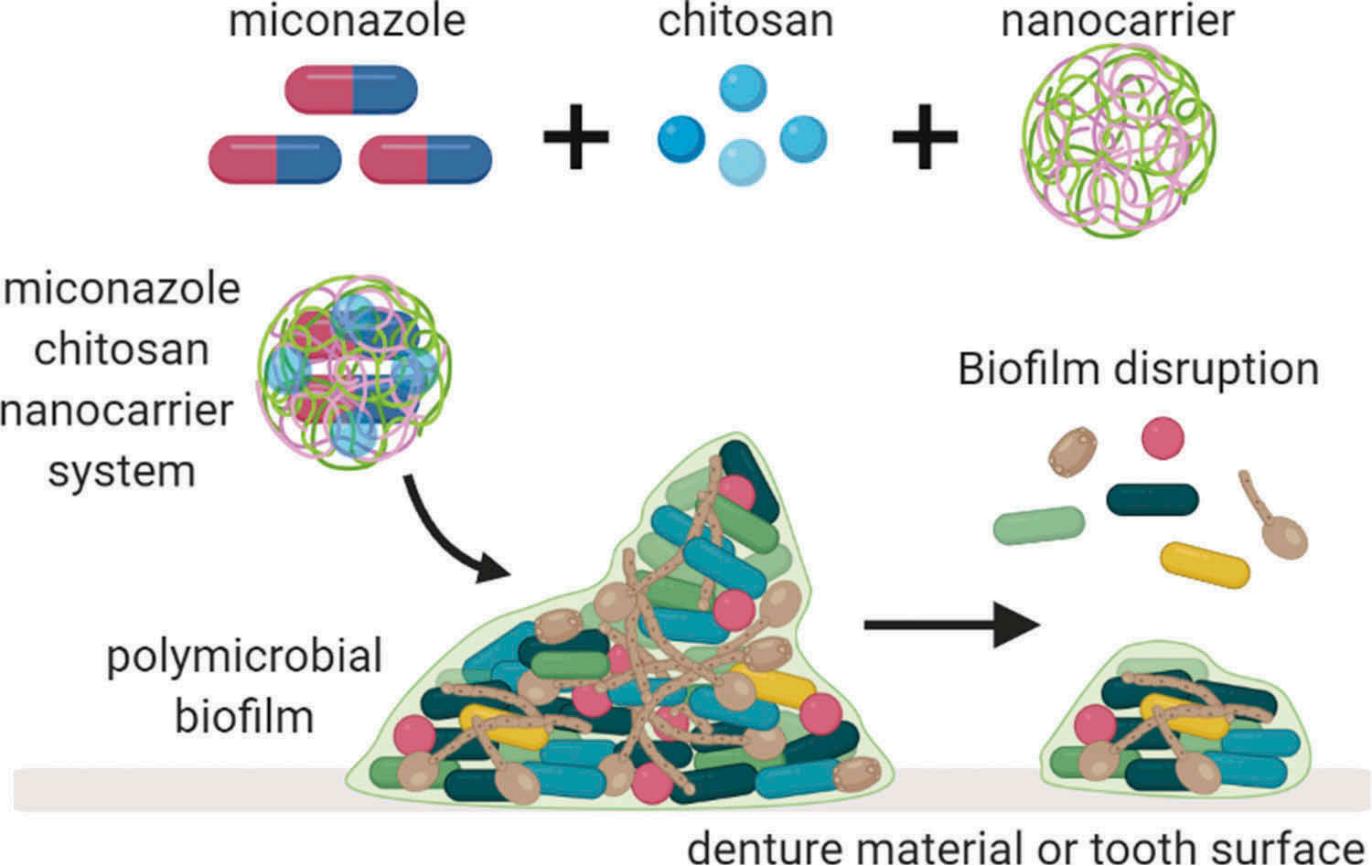
**Graphical representation depicting the nanosystem effects on oral biofilms**. The nanosystem used in this study was comprised of IONPs colloidal suspension, chitosan combined with miconazole (MCZ) and tested against three oral disease biofilm models; gingivitis, denture and dental caries.

Our improved understanding that microorganisms form complex polymicrobial communities, and that interkingdom interactions play an important role within these, has paved the way to enhance and develop novel methods and strategies to manage complex biofilms infections [33,34]. For the development of new antimicrobials, we must be conscious of what we are attempting to treat. Therefore, models that accurately recapitulate the *in vivo* scenario are likely to yield breakthroughs in potential therapeutics. Here, we describe three relevant complex interkingdom oral biofilm models to test a novel nanocarrier system that incorporates MCZ with core-shell compound of IONPs-CS (Figure 7). We report for the first time the effect of a MCZ nanocarrier against three different pathogenic biofilm models, first evaluating the MICs of MCZ alone and IONPs-CS-MCZ for all studied microorganisms in planktonic form and mono-species biofilms, before assessing the changes in number of cells, biofilm ultrastructure and species composition for the polymicrobial biofilm models following treatment with the nanocarrier system.

The results of planktonic and sessile MICs of MCZ and IONPs-CS-MCZ against several *C. albicans* strains showed that the planktonic cells were equally affected by the tested drugs. However, according to the sMIC_80_ results, it was possible to obtain higher antimicrobial effect at lower concentrations of MCZ against biofilms, by using a nanocarrier. Miconazole has been used for over 30 years and is prescribed specifically against oral fungal infections for topical use [35,36]. Different from other azoles, MCZ has a dual mechanism of action; besides interfering with ergosterol synthesis by inhibition of lanosterol demethylase, it also inhibits fungal catalase and peroxidase, therefore increasing intracellular reactive oxygen species and leading to cell death [37]. As for CS, literature suggests that the interaction between the positively charged drug and the negatively charged bacterial cell membranes results in the leakage of intracellular constituents [38,39]. Therefore, the lower MIC values found for the IONPs-CS-MCZ could be explained by the synergistic action between CS and MCZ or that drug-delivery nanosystems are designed to break through physical barriers to target the cell of interest [21,40]. Additional data from this study showed that IONPs-CS-MCZ was able to act effectively against planktonic and sessile cells of most microbial species used in the three biofilm models, confirming that it has both antifungal and antibacterial activity. Indeed, literature data reports the antibacterial potential of topical use concentrations of MCZ, specially against Gram-positive bacteria [16].

Previous work from our group has led to the development of three different biofilm models used in this study (gingivitis, denture and dental caries), taking into consideration early and later colonizers involved in the development of biofilms associated with the aforementioned oral diseases [4,27,28,30]. Since *C. albicans* is a structurally dominant cell of many polymicrobial biofilm-related diseases, it was specifically incorporated in all three biofilm models. In these models, reductions in the number of CFUs and metabolic activity for all polymicrobial biofilms were observed following treatment with MCZ, and generally reduced further following combination of MCZ with the nanosystem. In addition, total biomass for denture and dental caries biofilm models were corroborated by SEM observations, whereby less dense biofilms were visible in those treated with IONPs-CS-MCZ nanocarrier. From the data gathered for pMIC and sMICs of the nanosytem against *C. albicans* strains as mono-species, it could be postulated that the equilibrium of a complex system such as biofilm can be broken by attacking its main support system, in this case, the *Candida* cells. Tentatively, it could be proposed that bacterial cells were more exposed to nanocarrier action resulting from reductions in the number of hyphae. However, *Candida* numbers remained relatively stable in the multi-species biofilm following treatment with MCZ or IONPs-CS-MCZ, suggestive that the synergistic interactions between the bacteria and fungi may be protecting the latter from the antimicrobial activity of the drug. This has been described elsewhere, whereby bacteria such as *Streptococcus* and *Staphylococcus* spp. can confer protection to *Candida* against antimicrobials, and vice versa, when grown in mixed culture [10,41,42].

For all biofilm models, a significant change in the predominance of the different species after treatment with the MCZ nanocarrier was observed. In general, the most prevalent species in all biofilm models were aerobic and anaerobic-facultative bacteria, such as *Streptococcus* spp., *Veillonella* spp. and *Actinomyces* spp. After exposure to MCZ only and IONPs-CS-MCZ, it becomes noticeable that proportionally the reduction in the number of most bacterial cells was much higher than that noted for *C. albicans*; which increased in percentage of the final composition in all models. A limitation of this study was the inability to distinguish between the numbers of yeast and hyphal *Candida* cells using the CFU and CFE counts. SEM analyses highlighted that fewer hyphae were present in the nanocarrier system-treated biofilms compared to controls, which may suggest that the treatment inhibited the ability of *Candida* to form and/or maintain hyphae. Therefore, the reduction in bacterial counts may be explained by the role of *Candida* in providing structural support for the bacterial community. The *Candida* yeast cell form has around 6.6 µm diameter, but its hyphae are much larger in size [43] and therefore serve as a scaffold for the smaller bacterial cells to attach [6]. Loss of this support structure may have resulted in the observed reductions in bacterial cell numbers.

Interestingly, for all biofilm models, *Streptococcus* spp. appeared to be most affected by the MCZ nanocarrier. This was particularly evident for the dental caries biofilm model. These results suggest that MCZ-based formulations may be an interesting method of treatment for polymicrobial biofilms that contain recognized microbial synergistic associations. For example, in the caries biofilm model, *S. mutans*-derived α-glucans surrounding the fungal cells could provide an additional ‘drug capture matrix’ that prevented uptake of antifungals, therefore reducing *Candida* cell death [44]. These interactions between *Candida* and bacteria are largely reported, especially *Candida-Streptococcus* interactions. For example, *C. albicans* can promote streptococci proliferation by providing suitable environmental conditions [8]. Conversely, some *Streptococcus spp*. such as *S. mutans* can produce glucosyltranferases that bind to the fungal surface. In addition, *S. mutans* generates extracellular polysaccharides (EPS) in the presence of sucrose, provide binding site for *S. mutans* while allowing *C. albicans* to adhere and colonize abiotic surfaces [7,9]. Interestingly, in the caries model the CFE counts for *Candida* showed no change following MCZ- or MCZ-nanocarrier system treatment. It could be postulated that *S. mutans*-derived carbohydrates is protecting *Candida* from the MCZ effects.

In general, IONPs-CS-MCZ nanocarrier showed a great versatility since it was able to reduce the number of viable cells in all the three pathogenic biofilm models tested, as well as the ability to promote changes in biofilm composition. This suggests that the nanocarrier may have the potential to fight important oral diseases (gingivitis, caries and denture stomatitis) associated with polymicrobial interkingdom biofilms formed by microbial species that establish beneficial interactions with each other. Nevertheless, future studies should be conducted to evaluate the effect of the nanocarrier on microcosm biofilms, as well as tests that explore the magnetic properties of the nanoparticles on improving drug delivery accuracy. These analyses will bring new knowledge about the antibiofilm effect of the IONPs-CS-MCZ nanocarrier which may favor the delivery of MCZ to the target cells, reducing the drug concentration used. Future studies most also consider conducting cytotoxicity tests on human cells if the nanocarrier system is to be considered as a potential therapeutic for clinical trials.

In summary, IONPs-CS-MCZ nanocarrier affected the composition of the three evaluated biofilms, causing high reduction of *Streptococcus* cells for all biofilms and enhanced proportion of *V. dispar, A. naeslundii* and *C. albicans* in the gingivitis, denture and dental caries models, respectively. This alteration was directly related to the antibiofilm effect of this compound possibly arising from reductions of *C. albicans* numbers and loss of biofilm integrity. Whether such nanocarrier systems can be used *in vivo* remains to be seen; however, it is clear that such technology has promising potential to circumvent the physical barrier presented by polymicrobial biofilms to enhance drug delivery and efficacy.

## Supplementary Material

Supplemental Material

## References

[1] Frencken JE, Sharma P, Stenhouse L, et al. Global epidemiology of dental caries and severe periodontitis - a comprehensive review. J Clin Periodontol. 2017;44(Suppl 18):S94–S105. 28266116 10.1111/jcpe.12677

[2] Lourenco AG, Ribeiro AERA, Nakao C, et al. Oral Candida spp carriage and periodontal diseases in HIV-infected patients in Ribeirao Preto, Brazil. Rev Inst Med Trop Sao Paulo. 2017;59:e29. 28591257 10.1590/S1678-9946201759029 PMC5459536

[3] Nakajima M, Umezaki Y, Takeda S, et al. Association between oral candidiasis and bacterial pneumonia: A retrospective study. Oral Dis. 2020;26(1):234–237. 10.1111/odi.13216 31621985

[4] Ramage G, O’Donnell L, Sherry L, et al. Impact of frequency of denture cleaning on microbial and clinical parameters - a bench to chairside approach. J Oral Microbiol. 2019;11(1):1538437. 30598732 10.1080/20002297.2018.1538437 PMC6225516

[5] Brown JL, Johnston W, Delaney C, et al. Polymicrobial oral biofilm models: simplifying the complex. J Med Microbiol. 2019;68(11):1573–15. 31524581 10.1099/jmm.0.001063

[6] Delaney C, Kean R, Short B, et al. Fungi at the Scene of the Crime: innocent Bystanders or Accomplices in Oral Infections? Curr Clin Microbiol Rep. 2018;5(3):190–200.

[7] Koo H, Andes DR, Krysan DJ. Candida-streptococcal interactions in biofilm-associated oral diseases. PLoS Pathog. 2018;14(12):e1007342. 30543717 10.1371/journal.ppat.1007342 PMC6292568

[8] Nobbs AH, Jenkinson HF. Interkingdom networking within the oral microbiome. Microbes Infect. 2015;17(7):484–492. 25805401 10.1016/j.micinf.2015.03.008 PMC4485937

[9] Xu H, Jenkinson HF, Dongari-Bagtzoglou A. Innocent until proven guilty: mechanisms and roles of Streptococcus-Candida interactions in oral health and disease. Mol Oral Microbiol. 2014;29(3):99–116. 24877244 10.1111/omi.12049 PMC4238848

[10] Kean R, Rajendran R, Haggarty J, et al. Candida albicans Mycofilms Support Staphylococcus aureus Colonization and Enhances Miconazole Resistance in Dual-Species Interactions. Front Microbiol. 2017;8:258. 28280487 10.3389/fmicb.2017.00258 PMC5322193

[11] Sztukowska MN, Dutton LC, Delaney C, et al. Community Development between Porphyromonas gingivalis and Candida albicans Mediated by InlJ and Als3. mBio. 2018;9(2):e00202-18. 10.1128/mBio.00202-18 PMC5915736 29691333

[12] Wu T, Cen L, Kaplan C, et al. Cellular Components Mediating Coadherence of Candida albicans and Fusobacterium nucleatum. J Dent Res. 2015;94(10):1432–1438. 26152186 10.1177/0022034515593706 PMC4577983

[13] Wistrand-Yuen E, Knopp M, Hjort K, et al. Evolution of high-level resistance during low-level antibiotic exposure. Nat Commun. 2018;9(1):1599. 29686259 10.1038/s41467-018-04059-1 PMC5913237

[14] Aslani N, Abastabar M, Hedayati MT, et al. Molecular identification and antifungal susceptibility testing of Candida species isolated from dental plaques. J Mycol Med. 2018;28(3):433–436. 29805065 10.1016/j.mycmed.2018.05.006

[15] Sardi JC, Almeida AM, Mendes Giannini MJ. New antimicrobial therapies used against fungi present in subgingival sites–a brief review. Arch Oral Biol. 2011;56(10):951–959. 21676377 10.1016/j.archoralbio.2011.03.007

[16] Nenoff P, Koch D, Krüger C, et al. New insights on the antibacterial efficacy of miconazole in vitro. Mycoses. 2017;60(8):552–557. 28370366 10.1111/myc.12620

[17] Miranda-Cadena K, Marcos-Arias C, Mateo E, et al. Prevalence and antifungal susceptibility profiles of Candida glabrata, Candida parapsilosis and their close-related species in oral candidiasis. Arch Oral Biol. 2018;95:100–107. 30096698 10.1016/j.archoralbio.2018.07.017

[18] Cross EW, Park S, Perlin DS. Cross-Resistance of clinical isolates of Candida albicans and Candida glabrata to over-the-counter azoles used in the treatment of vaginitis. Microb Drug Resist. 2000;6(2):155–161. 10990271 10.1089/107662900419474

[19] Müller FM, Weig M, Peter J, Walsh TJ. Azole cross-resistance to ketoconazole, fluconazole, itraconazole and voriconazole in clinical Candida albicans isolates from HIV-infected children with oropharyngeal candidosis. J Antimicrob Chemother. 2000;46(2):338‐340. 10.1093/jac/46.2.338 10933673

[20] Patra JK, Das G, Fraceto LF, et al. Nano based drug delivery systems: recent developments and future prospects. J Nanobiotechnology. 2018;16(1):71. 30231877 10.1186/s12951-018-0392-8 PMC6145203

[21] Arias LS, Hardick J, Barnes M, et al. Iron Oxide Nanoparticles for Biomedical Applications: A Perspective on Synthesis, Drugs, Antimicrobial Activity, and Toxicity. Antibiotics (Basel). 2018;7(2). DOI:10.3390/antibiotics7030077. PMC6023022 29890753

[22] Vieira APM, Arias LS, de Souza Neto FN, et al. Antibiofilm effect of chlorhexidine-carrier nanosystem based on iron oxide magnetic nanoparticles and chitosan. Colloids Surf B Biointerfaces. 2019;174:224–231. 30465997 10.1016/j.colsurfb.2018.11.023

[23] Nehra P, Chauhan RP, Garg N, et al. Antibacterial and antifungal activity of chitosan coated iron oxide nanoparticles. Br J Biomed Sci. 2018;75(1):13–18. 28945174 10.1080/09674845.2017.1347362

[24] Arias LS, Pessan JP, de Souza Neto FN, et al. Novel nanocarrier of miconazole based on chitosan-coated iron oxide nanoparticles as a nanotherapy to fight Candida biofilms. Colloids Surf B Biointerfaces. 2020:111080. DOI:10.1016/j.colsurfb.2020.111080. 32361504

[25] Allkja J, Bjarnsholt T, Coenye T, et al. Minimum information guideline for spectrophotometric and fluorometric methods to assess biofilm formation in microplates. Biofilm. 2020;2. DOI:10.1016/j.bioflm.2019.100010. PMC7798448 33447797

[26] Miles AA, Misra SS, Irwin JO. The estimation of the bactericidal power of the blood. J Hyg (Lond). 1938;38(6):732–749. 10.1017/s002217240001158x PMC2199673 20475467

[27] Zhou Y, Millhouse E, Shaw T, et al. Evaluating Streptococcus mutans Strain Dependent Characteristics in a Polymicrobial Biofilm Community. Front Microbiol. 2018;9:1498. 30083138 10.3389/fmicb.2018.01498 PMC6064717

[28] Brown JL, Johnston W, Delaney C, et al. Biofilm-stimulated epithelium modulates the inflammatory responses in co-cultured immune cells. Sci Rep. 2019;9(1):15779. 31673005 10.1038/s41598-019-52115-7 PMC6823452

[29] Montelongo-Jauregui D, Srinivasan A, Ramasubramanian AK, et al. An In Vitro Model for Oral Mixed Biofilms of Candida albicans and Streptococcus gordonii in Synthetic Saliva. Front Microbiol. 2016;7:686. 27242712 10.3389/fmicb.2016.00686 PMC4864667

[30] Sherry L, Lappin G, O’Donnell LE, et al. Viable Compositional Analysis of an Eleven Species Oral Polymicrobial Biofilm. Front Microbiol. 2016;7:912. 27375612 10.3389/fmicb.2016.00912 PMC4902011

[31] Erlandsen SL, Kristich CJ, Dunny GM, et al. High-resolution visualization of the microbial glycocalyx with low-voltage scanning electron microscopy: dependence on cationic dyes. J Histochem Cytochem. 2004;52(11):1427–1435. 15505337 10.1369/jhc.4A6428.2004 PMC3957825

[32] Tunney MM, Patrick S, Gorman SP, et al. Improved detection of infection in hip replacements. A currently underestimated problem. J Bone Joint Surg Br. 1998;80(4):568–572. 9699813 10.1302/0301-620x.80b4.8473

[33] Allison DL, Willems HME, Jayatilake JAMS et al. Candida-Bacteria Interactions: their Impact on Human Disease. Microbiol Spectr. 2016;4(3). 10.1128/microbiolspec.VMBF-0030-2016 27337476

[34] Ramage G, Rajendran R, Sherry L, et al. Fungal biofilm resistance. Int J Microbiol. 2012;2012:528521. 22518145 10.1155/2012/528521 PMC3299327

[35] Garcia-Cuesta C, Sarrion-Perez MG., Bagan JV. Current treatment of oral candidiasis: A literature review. J Clin Exp Dent. 2014;6(5):e576–82. 25674329 10.4317/jced.51798 PMC4312689

[36] Brincker H. Prophylactic treatment with miconazole in patients highly predisposed to fungal infection. A placebo-controlled double-blind study. Acta Med Scand. 1978;204(1–2):123–128. 356523 10.1111/j.0954-6820.1978.tb08410.x

[37] Delattin N, Cammue BP, Thevissen K. Reactive oxygen species-inducing antifungal agents and their activity against fungal biofilms. Future Med Chem. 2014;6(1):77–90. 24358949 10.4155/fmc.13.189

[38] Lou MM, Zhu B, Muhammad I, et al. Antibacterial activity and mechanism of action of chitosan solutions against apricot fruit rot pathogen Burkholderia seminalis. Carbohydr Res. 2011;346(11):1294–1301. 21605851 10.1016/j.carres.2011.04.042

[39] Rabea EI, Badawy ME-T, Stevens CV, et al. Chitosan as antimicrobial agent: applications and mode of action. Biomacromolecules. 2003;4(6):1457–1465. 14606868 10.1021/bm034130m

[40] Rajkumar S, Prabaharan M. Multi-functional nanocarriers based on iron oxide nanoparticles conjugated with doxorubicin, poly(ethylene glycol) and folic acid as theranostics for cancer therapy. Colloids Surf B Biointerfaces. 2018;170:529–537. 29966906 10.1016/j.colsurfb.2018.06.051

[41] Harriott MM, Noverr MC. Candida albicans and Staphylococcus aureus form polymicrobial biofilms: effects on antimicrobial resistance. Antimicrob Agents Chemother. 2009;53(9):3914–3922. 19564370 10.1128/AAC.00657-09 PMC2737866

[42] Xu H, Sobue T, Thompson A, et al. Streptococcal co-infection augments Candida pathogenicity by amplifying the mucosal inflammatory response. Cell Microbiol. 2014;16(2):214–231. . 24079976 10.1111/cmi.12216 PMC3956708

[43] Mukaremera L, Lee KK, Mora-Montes HM, et al. Candida albicans Yeast, Pseudohyphal, and Hyphal Morphogenesis Differentially Affects Immune Recognition. Front Immunol. 2017;8:629. 28638380 10.3389/fimmu.2017.00629 PMC5461353

[44] Kim D, Liu Y, Benhamou RI, et al. Bacterial-derived exopolysaccharides enhance antifungal drug tolerance in a cross-kingdom oral biofilm. Isme J. 2018;12(6):1427–1442. 29670217 10.1038/s41396-018-0113-1 PMC5955968

