# Supplementary material for: A nanocarrier system that potentiates the effect of miconazole within different interkingdom biofilms
Source: J Oral Microbiol. 2020 Jun 7;12(1):1771071. doi: 10.1080/20002297.2020.1771071 (PMC7448886; doi:10.1080/20002297.2020.1771071)
Supplement: Supplemental Material [file ZJOM_A_1771071_SM1972.docx]

**A nanocarrier system potentiates the effect of miconazole on three *in vitro* oral models of polymicrobial biofilms**

Laís Salomão Arias^1^, Jason L Brown^2^, Mark C Butcher^2^, Christopher Delaney^2^, Douglas Roberto Monteiro^1,3^, Gordon Ramage^2*^

**Supplementary Files**

**Supplementary Table 1.** Mean CFE/ml values for each microorganism from the three different biofilm models (gingivitis, denture and dental caries). Mean values taken from six colony forming equivalent values for all microorganisms in the three models (e.g., three technical replicates from two experiments).

|  | |  | Gingivitis model | | | Denture model | | | Dental caries model | | |
| --- | --- | --- | --- | --- | --- | --- | --- | --- | --- | --- | --- |
|  | |  | Control | MCZ | Nanocarrier | Control | MCZ | Nanocarrier | Control | MCZ | Nanocarrier |
|  | Total CFE/mL | | 2.1 x 10^9^ | 4.4 x 10^8^ | 3.1 x 10^8^ | 6.8 x 10^8^ | 5.5 x 10^7^ | 6.4 x 10^6^ | 5.9 x 10^8^ | 1.4 x 10^8^ | 6.2 x 10^7^ |
| Mean CFE/mL | *Candida* | | 2.25x10^6^ | 1.22x10^6^ | 1.09x10^6^ | 1.56x10^7^ | 7.94x10^6^ | 4.16x10^5^ | 2,73x10^7^ | 1.57x10^7^ | 2.18x10^7^ |
|  | Streptococci | | 9.68x10^8^ | 2.08x10^8^ | 4.82x10^7^ | 3.39x10^8^ | 5.83x10^6^ | 5.77x10^5^ | 1.02x10^8^ | 7.67x10^7^ | 4.99x10^5^ |
|  | *V. dispar* | | 5.73x10^8^ | 1.85x10^8^ | 1.36x10^8^ | 1.77x10^8^ | 1.19x10^7^ | 3.17x10^6^ | 2.65x10^8^ | 3.66x10^7^ | 2.06x10^7^ |
|  | *A. naeslundii* | | 5.12x10^8^ | 2.47x10^8^ | 1.27x10^8^ | 1.44x10^8^ | 1.33x10^7^ | 3.72x10^6^ | 1.87x10^8^ | 1.07x10^7^ | 8.11x10^6^ |
|  | *Fusobacterium* | | 4.48x10^7^ | 2.05x10^6^ | 6.20x10^5^ | n/a  _______ | n/a | n/a | 3.42x10^7^ | 7.74x10^5^ | 2.36x10^4^ |
|  | *L. casei* | | n/a | n/a | n/a | 6.02x10^6^ | 1.32x10^7^ | 1.13x10^6^ | 6.50x10^6^ | 1.06x10^7^ | 4.77x10^6^ |
|  | *L. zeae* | | n/a | n/a | n/a | 2.78x10^6^ | 2.80x10^6^ | 2.43x10^5^ | n/a | n/a | n/a |
|  | *R. dentocariosa* | | n/a | n/a | n/a | 6.51x10^5^ | 1.16x10^4^ | 9.67x10^3^ | n/a | n/a | n/a |

**
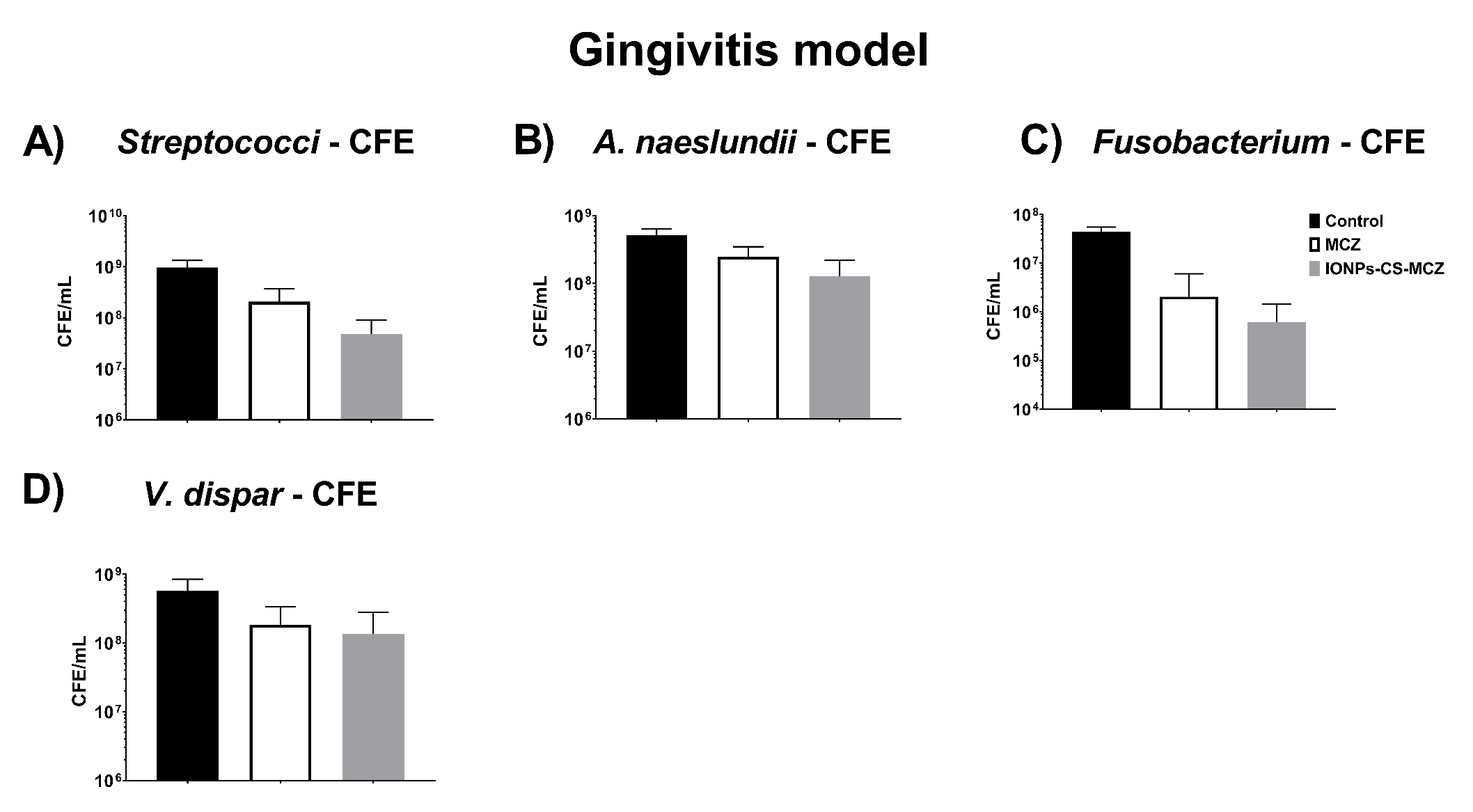
**

**Supplementary Figure 1.** Viable colony forming equivalents for each microorganism in the gingivitis biofilm +/- nanocarrier system with miconazole. Colony forming equivalents for each microorganism (Streptococci spp., A; *A. naeslundii*, B; *Fusobacterium* spp., C and *V. dispar*, D) in the gingivitis biofilm models were calculated from qPCR analyses. Gingivitis biofilms were treated with MCZ only or IONPs-CS-MCZ (miconazole; 64mg/L) for 24 h~~ours~~ anaerobically prior to DNA extraction and qPCR analysis. Untreated biofilms were cultured with media only for 24 h~~ours~~. Results shown representative of a total of six values (three technical replicates from two separate experiments)

**
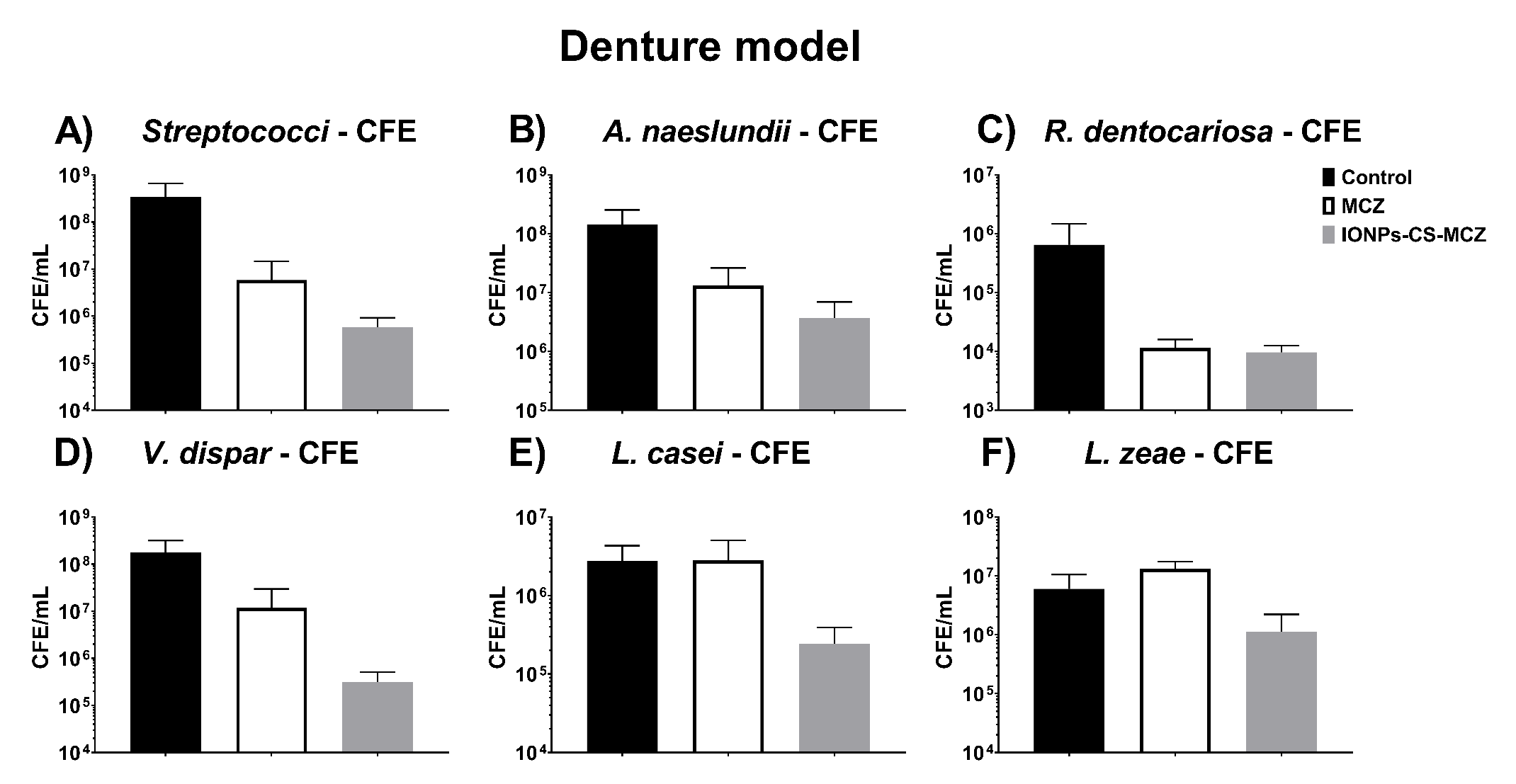
**

**Supplementary Figure 2.** Colony forming equivalents of viable cells of all bacteria in the denture biofilm model with or without nanocarrier treatment. Colony forming equivalents for each microorganism (Streptococci spp., A; *A. naeslundii*, B; *R. denticariosa*, C and *V. dispar*, D; *L. casei*, E; *L. zeae*, F) in the denture biofilm models were calculated from qPCR analyses. Biofilms were treated in media +/- MCZ only or IONPs-CS-MCZ (miconazole; 64mg/L), anaerobically for 24 h~~ours~~. DNA was extracted from treated and untreated biofilms prior to qPCR analyses. Results shown representative of a total of six values (three technical replicates from two separate experiments).


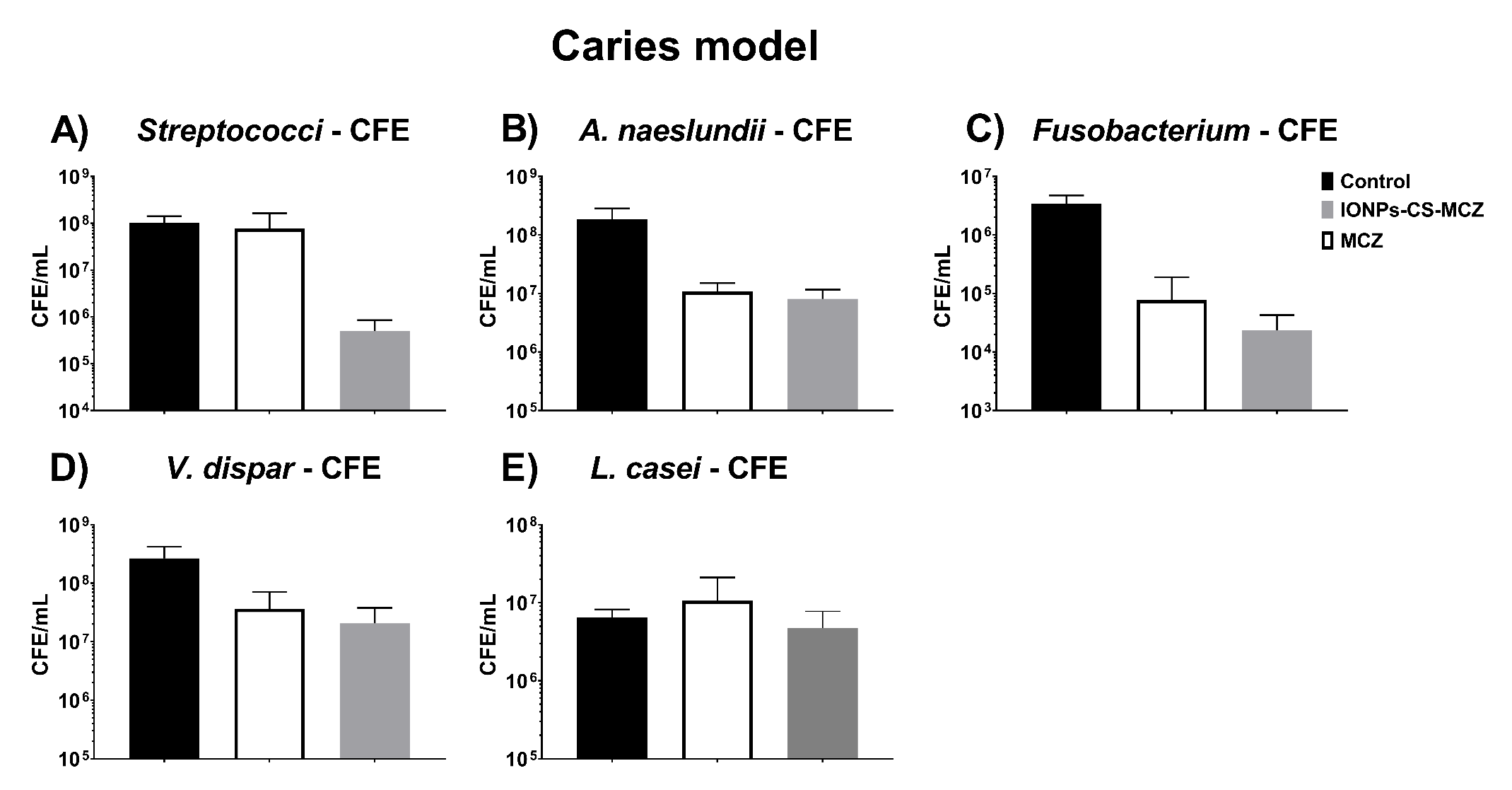


**Supplementary Figure 3.** Colony forming equivalents of viable cells of all bacteria in the caries biofilm model treated with miconazole nanocarrier system. Colony forming equivalents for each microorganism (Streptococci spp., A; *A. naeslundii*, B; *R. denticariosa*, C; *Fusobacterium* spp., D; *V. dispar*, E; *L. casei*, F) in the caries biofilm models were calculated from qPCR analyses. Biofilms were treated with or without nanocarrier system (miconazole; 64mg/L). DNA was extracted from treated and untreated biofilms prior to qPCR analyses, and CFE counts calculated from standard curves generated for each microorganism. Results shown representative of a total of six values (three technical replicates from two separate experiments).
